# The crosstalk between nitrate signaling and other signaling molecules in *Arabidopsis thaliana*

**DOI:** 10.3389/fpls.2025.1546011

**Published:** 2025-03-10

**Authors:** Jingjing Mao, Zhen Tian, Jinhao Sun, Duanfei Wang, Yating Yu, Shaopeng Li

**Affiliations:** Technology Centre, China Tobacco Jiangsu Industrial Co., Ltd., Nanjing, China

**Keywords:** NUE, nitrate signaling, phytohormones, ROS, calcium, peptides, sucrose, crosstalk

## Abstract

Nitrate signaling coordinates the expression of a broad range of genes involved in nitrate uptake, transport, and assimilation, playing a crucial role in plant growth and development. Notably, nitrate signaling interacts extensively with various messenger molecules, including phytohormones, calcium ions (Ca^2+^), reactive oxygen species (ROS), peptides, and sucrose. This crosstalk amplifies nitrate signaling and optimizes nutrient uptake, coordinating developmental processes and enhancing stress tolerance. Understanding the interactions between nitrate and these signaling molecules offers valuable insights into improving crop nutrient use efficiency (NUE), stress resilience, and agricultural sustainability. Using *Arabidopsis thaliana* as a model, this review consolidates current knowledge on nitrate signaling and its interplay with other signaling pathways that regulate plant development and adaptation. Finally, the review highlights potential genetic strategies for enhancing NUE, contributing to more sustainable agricultural practices.

## Introduction

Nitrate (NO_3_^−^) is the primary source of nitrogen, an essential element for plants to form key biomolecules like amino acids, proteins, and nucleic acids. In addition to its nutritional role, nitrate also serves as a signaling molecule that regulates plant growth, development, and responses to environmental factors. However, excessive nitrate use in crops with low nitrogen use efficiency (NUE) has led to higher fertilizer costs and environmental issues like nitrate leaching, which causes eutrophication (Yadav et al., 2017; Bijay and Craswell, 2021).

NUE refers to the ability of a plant to utilize available nitrogen for growth and production effectively. It measures how efficiently plants can absorb, assimilate, and utilize nitrogen, especially in relation to the amount of nitrogen provided, such as through fertilizers. NUE is a crucial factor in enhancing agricultural productivity and promoting sustainability in farming practices. One promising strategy to improve NUE is the genetic manipulation of nitrate signaling pathways. Modern molecular breeding techniques, such as marker-assisted selection, genomic selection, and genome-wide association studies, facilitate the identification and selection of genes linked to NUE. Genetic engineering contributes to improving NUE by introducing novel genes or pathways to enhance nitrogen metabolism. In addition, breakthroughs in genome editing technologies provide the possibility to precisely modify genes involved in nitrate signaling. Therefore, a thorough understanding of nitrate signaling and its integration with other cellular networks is crucial for the improvement of NUE *via* genetic manipulation.

Advances in the model plant *Arabidopsis thaliana* have provided valuable insights into the molecular mechanisms underlying nitrate signaling and its regulatory processes. Central to nitrate signaling are key events such as nitrate uptake, root-to-shoot transport, allocation within plant tissues, and cellular transport. Many nitrate transporter are involved in these processes, which have been extensively reviewed (Wang et al., 2018b; Islam et al., 2020; Vidal et al., 2020). This signaling pathway is not only regulated at both the transcriptional and post-transcriptional levels but also undergoes extensive crosstalk with other signaling molecules, including phytohormones, calcium ions (Ca^2+^), reactive oxygen species (ROS), peptides, and sucrose. The interaction with these molecules amplifies nitrate signaling, optimizing nutrient uptake and enhancing plant resilience by remodeling root architecture and shoot growth, highlighting the integrated nature of nutrient signaling in plant physiology. A comprehensive understanding of nitrate signaling and its interactions with other cellular networks is essential for advancing both fundamental plant biology and agricultural applications. This review aims to enhance our understanding of these signaling interactions in Arabidopsis, offering insights that could improve crop NUE and resilience in agricultural systems. We will also explore potential strategies to leverage this knowledge for enhancing NUE in crops.

## Phytohormones

**Figure 1:**
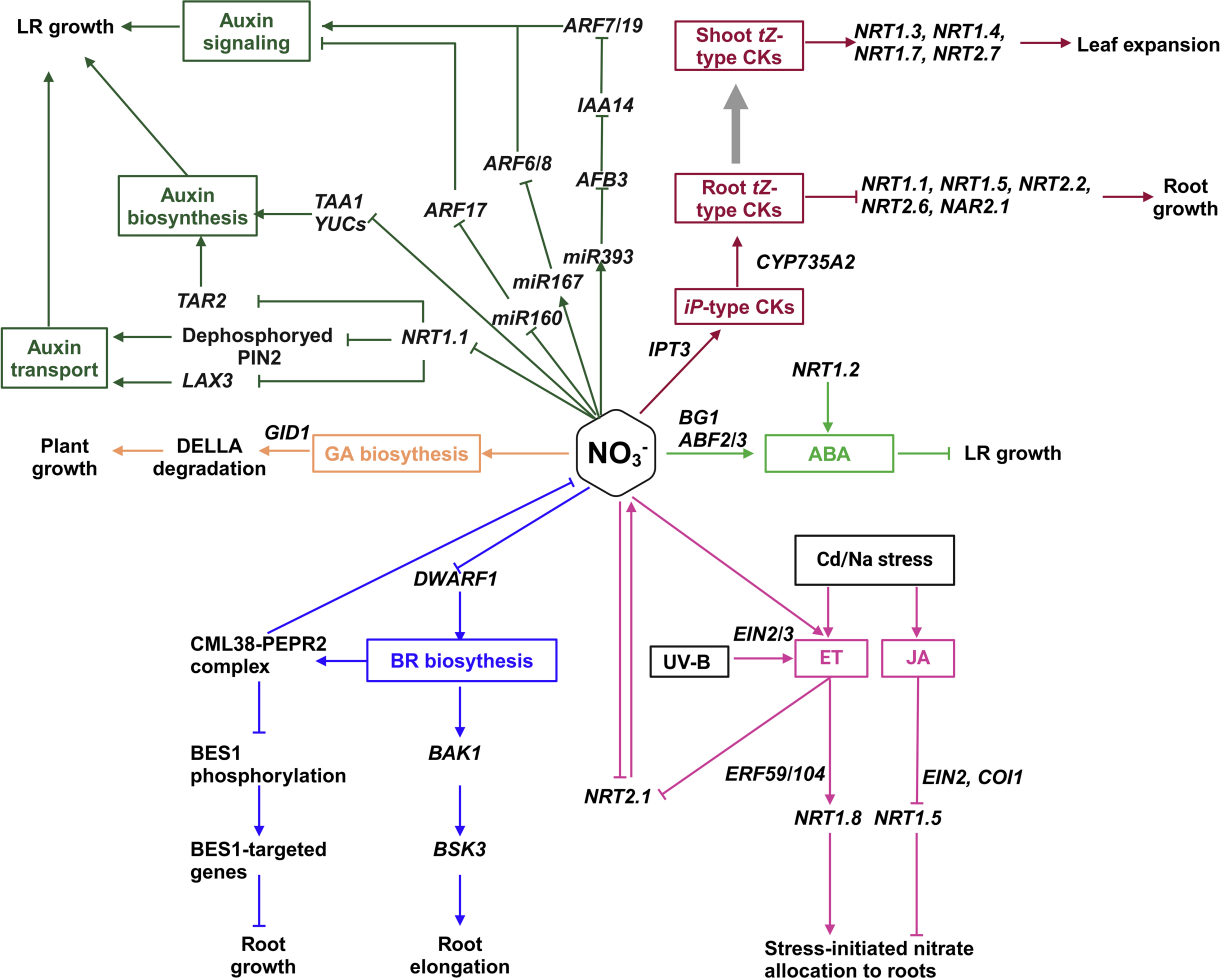
Illustration of the crosstalk between nitrate signaling and phytohormone signaling. Pointed and blunt arrowheads indicate activating and inhibiting interactions, respectively. The thick arrow indicates the root-to-shoot translocation.

Environmental nitrate levels fluctuate across space and time, requiring plants to dynamically adjust root and shoot growth to optimize nitrate uptake and acquisition. Information about external nitrate availability and the overall demand of the plant is translated into cellular signals including phytohormones (illustrated in **Figure 1**). The crosstalk between phytohormones and nitrate signaling is mainly reflected in the developmental plasticity of root system architecture (RSA), referred to as root foraging, which enables plants to efficiently seek out nutrient pools that fluctuate spatially and temporally (illustrated in **Figure 1**). RSA includes the initiation, emergence, and elongation of primary roots (PR), lateral roots (LR), as well as root hairs (HR) (Jia et al., 2022).

### Auxin

The basipetal transport of auxin has long been recognized as a crucial mechanism for transmitting nitrate signals from the shoot to the root, driving the remodeling of RSA (Li, 2024). Nitrate deficiency directly affects auxin transport, with the key nitrate transporter NRT1.1 (Nitrate Transporter 1.1) playing a pivotal role in this process. Notably, NRT1.1 is also identified as an auxin influx facilitator, contributing to auxin transport activity modulated by nitrate (Krouk et al., 2010). Under low nitrate conditions, NRT1.1 prevents auxin accumulation in the root tip, thereby inhibiting LR growth. In contrast, when nitrate levels are high, the auxin transport activity of NRT1.1 is inhibited, leading to auxin accumulation in the root tip and promoting LR outgrowth (Krouk et al., 2010). In the absence of nitrate, NRT1.1 also negatively regulates the auxin influx carrier *LAX3* (*Like-Aux1-3*), reducing auxin levels in the LR and impeding its development (Ma et al., 2014; Maghiaoui et al., 2020). Interestingly, NRT1.1 is essential for modulating protein phosphorylation in response to nitrate. In fact, only 4% of the nitrate-phosphoproteome observed in wild-type plants was retained in the *nrt1.1* mutant (Vega et al., 2021). The dephosphorylation of the Ser_439_ residue in PIN2 (PIN-Formed 2), an auxin efflux carrier, promotes its polar localization in the plasma membrane and modulates auxin flow between adjacent tissues, thereby inhibiting both PR and LR elongation (Vega et al., 2021). Additionally, the auxin transport of PIN7, another auxin efflux carrier, is regulated by NRT2.1 through direct interaction. The *nrt2.1* mutant exhibits defects in polar auxin transport, resulting in impaired low-nitrate-mediated suppression of PR growth (Wang et al., 2023).

NRT1.1 also negatively regulates the auxin biosynthetic gene *TAR2* (*Arabidopsis* Tryptophan Aminotransferase-Related 2), which encodes a key enzyme in the tryptophan-dependent auxin biosynthesis pathway, thereby inhibiting LR development (Ma et al., 2014; Maghiaoui et al., 2020). In addition, nitrate deficiency induces the expression of auxin biosynthesis genes *YUC3/5/7/8* and *TAA1* (Tryptophan Aminotransferase of Arabidopsis 1), leading to increased auxin accumulation at LR tips and influencing root elongation (Jia et al., 2021). It has been shown that RH elongation under nitrogen deficiency relies on TAA1- and YUC8-mediated auxin biosynthesis at the root apex, alongside AUX1 (Auxin Transporter Protein 1)- and PIN2-driven auxin transport toward the shoots (Jia et al., 2023). In the RH zone, auxin transport driven by AUX1 and PIN2 activates the transcription factors ARF6/8 (Auxin Response Factor), thereby promoting RH elongation in response to low nitrogen availability (Jia et al., 2023).

Furthermore, nitrate availability plays a role in auxin signaling by post-transcriptionally regulating related genes. ARF6/8 are the cleavage targets of miR167, a microRNA that promotes mRNA degradation or inhibits translation of its targets (Wu et al., 2006). Under nitrogen deficiency, the expression of *miR167* is repressed, thereby relieving its inhibition on *ARF8* transcripts, which in turn promotes LR outgrowth and adventitious roots (Gutierrez et al., 2009; Liang et al., 2012). miR160, another microRNA induced under nitrogen deficiency, targets the transcripts of *ARF10*, *ARF16*, and *ARF17* for degradation, facilitating adventitious root outgrowth by repressing *ARF17* (Wang et al., 2005; Gutierrez et al., 2009; Liang et al., 2012). Additionally, *miR393* and its cleavage target *AFB3* (*Auxin Signaling F-box 3*) are both nitrate-induced. In addition, both *afb3* mutants and *miR393*-overexpressing lines exhibited a decreased density of emerging and initiating LR compared to wild-type under KNO_3_ treatment (Vidal et al., 2010). These suggest that the miR393-AFB3 module forms a feed-forward mechanism to regulate RSA in response to nitrogen availability. AFB3 is also found to mediate the degradation of the transcriptional repressor IAA14 (Indole-3-Acetic Acid Inducible 14) to effectively alleviate the repression exerted on *ARF7* and *ARF19*, promoting auxin signaling and LR initiation (Okushima et al., 2005).

### Cytokinins

CKs are shown to act as long-distance signaling molecules that move between root and shoot. This translocation is facilitated by the xylem transport system driven by transpiration flow and the phloem transport system that delivers photosynthate throughout the body of the plant (Kudo et al., 2010). CKs have been shown to repress all root-specific root-type *NRT* genes (*NRT1.1*, *NRT1.5*, *NRT2.1*, *NRT2.2*, *NRT2.6*, and *NAR2.1*), an effect that is independent of the nitrogen status of plants, whether under high or low nitrate conditions (Kiba et al., 2011). This suggests that CKs function as a nitrogen satiety signal, inhibiting nitrate uptake in the root. In addition to this repressive role, CKs positively regulate certain *NRT* genes in the shoot. Specifically, shoot-expressed *NRT* genes (*NRT1.3*, *NRT1.4*, *NRT1.7*, *NRT2.7*) are up-regulated by CKs under both high nitrate and low nitrate conditions, indicating that CKs may enhance nitrate distribution and translocation within shoots (Kiba et al., 2011).

In verse, nitrate supply induces CK accumulation, which may subsequently travel through the vascular bundles (Sharma and Zheng, 2019; Li et al., 2021). Studies have shown that nitrate upregulates the expression of *IPT3* (*Isopentenyl Transferase 3*), a phloem companion cell-localized gene encoding adenosine phosphates-isopentenyltransferase, an enzyme that catalyzes the initial step of cytokinin biosynthesis (Takei et al., 2004a; Hirose et al., 2008). Similarly, nitrate enhances the expression of *CYP735A2* (*Cytochrome P450 735A2*), a vascular bundle-localized gene encoding cytokinin hydroxylase, which catalyzes the biosynthesis of *trans-zeatin* (tZ) cytokinin (Takei et al., 2004b). Nitrate feeding is proposed to stimulate the synthesis of *isopentenyl (iP)*-type CK in root phloem after IPT3 activation, with CYP735A2 subsequently converting these into *trans-zeatin* (tZ) (Hirose et al., 2008; Ahmad et al., 2023). This nitrate-induced accumulation of CK regulates both root growth and leaf expansion through root-to-shoot transport.

### ABA

The link between nitrate and ABA was first identified nearly two decades ago, highlighting the role of ABA in inhibiting LR growth under high nitrate conditions (Signora et al., 2001). In WT plants, high nitrate levels (10 mM) reduced LR formation compared to low nitrate conditions (0.1 mM). In contrast, ABA biosynthesis and ABA-insensitive mutants (*abi4* and *abi5*) displayed a similar number of LR regardless of nitrate concentration (Signora et al., 2001), suggesting that ABA mediates the inhibition of LR growth under elevated nitrate levels. Interestingly, although NRT1.2 functions as both an ABA and nitrate transporter, its ABA transport activity remains unaffected by high nitrate concentrations, suggesting that the physiological linkage between nitrate and ABA signals may be not through NRT1.2 (Kanno et al., 2013). Subsequent studies demonstrated that nitrate induces the expression of the BG1 (Enzyme β-glucosidase 1), which releases ABA from ABA-glucose ester stores in the ER, promoting ABA accumulation in the root meristem and thereby modulating root growth (Ondzighi-Assoume et al., 2016). Besides, ABA Responsive Element Binding Factors 2 and 3 (ABF2 and ABF3) are key regulators of the endodermis response to nitrate, their targets account for more than 50% of the nitrate-responsive transcriptome in the endodermis (Contreras-Lopez et al., 2022). The positive effect of nitrate on LR growth was absent in *abf2*, *abf3*, and *abf2abf3* mutants, suggesting their roles in LR development in response to nitrate (Contreras-Lopez et al., 2022).

### Gibberellin

Nitrate supply enhances GA biosynthesis in *Arabidopsis* (Camut et al., 2021). GAs promote growth by opposing the functions of DELLA growth-repressing proteins (DELLAs), members of the GRAS family of transcription regulators (Davière and Achard, 2013). GA binds to the GA receptors Gibberellin Insensitive 1 (GID1) to trigger its degradation of DELLAs. Usually, DELLA accumulation reduces growth to prioritize resources for defense mechanisms, while GA-mediated DELLA degradation stimulates growth under favorable conditions (Colebrook et al., 2014). It was found that nitrate reduces the abundance of DELLAs by increasing GA contents through activation of GA metabolism gene expression (Camut et al., 2021). Consistent with that, the growth restraint under nitrate deficiency is partially rescued in mutants lacking all *DELLAs* (Camut et al., 2021).

### Brassinosteroids

BRs have been reported to enhance root foraging for nutrients under mild nitrogen deficiency conditions (Jia et al., 2019, Jia et al., 2020). Further studies revealed that nitrogen deficiency specifically upregulates the expression of the BR biosynthesis gene *DWARF1* as well as the BR co-receptor *BAK1* (*BRI1-Associated Receptor Kinase 1*) (Jia et al., 2019, Jia et al., 2020). BAK1 further activates downstream BSK3 (Brassinosteroid Signaling Kinase 3), a key protein involved in modulating root elongation (Jia et al., 2019). CML38 (Calmodulin-like-38) and PEPR2 (PEP1 Receptor 2) are induced by exogenous nitrate and BR, they interact at the cell membrane to regulate shared downstream genes involved in both the nitrate and BR signaling pathways. The CML38-PEPR2 complex not only transduces nitrate signals to reduce nitrate uptake and assimilation, but also mediates BR signaling by suppressing BES1 (BRI1-EMS-Suppressor 1) phosphorylation and upregulating *BES1*-target genes, ultimately inhibiting root growth. As such, it has been identified as a convergence module linking nitrate and BR signaling (Song et al., 2021). Under low-nitrogen conditions, mutants of these genes exhibited enhanced PR growth and increased LR development (Song et al., 2021).

### Ethylene and Jasmonic acid

ET production increases in *Arabidopsis* roots when shifted from low (0.1 mM) to high nitrate concentrations (10 mM) (Tian et al., 2009) or from high (10 mM) to low nitrate concentrations (0.2 mM) (Zheng et al., 2013), indicating enhanced ET biosynthesis during nitrate fluctuations in the root environment. At high nitrate concentrations, the expression of *NRT1.1* and *NRT2.1* is upregulated and downregulated, respectively. However, this regulatory response is absent in *etr1* (*Ethylene Resistant 1*) and *ein2* (*Ethylene Insensitive 2*) mutants, suggesting a role for ET in nitrate signaling (Tian et al., 2009). Under low-nitrate conditions, *NRT2.1* expression is induced to enhance high-affinity nitrate uptake, potentially lowering nitrate levels around the roots and subsequently stimulating ET biosynthesis. The increased ET then downregulates *NRT2.1* expression, forming a negative feedback loop (Zheng et al., 2013). These findings imply that ET may interact with nitrate signaling, particularly in response to or during recovery from nitrate deficiency.

Stressors trigger stress-initiated nitrate allocation to roots (SINAR), enhancing root-based nitrate assimilation. This process may be more energy-efficient, as it reduces competition with photosynthesis for reductants and energy, thereby supporting growth under adverse conditions (Zhang et al., 2014). SINAR levels are reduced in *ein2* and *coi1* (*Coronatine Insensitive 1*, which encodes a JA receptor) mutants, suggesting that it is primarily regulated by ET and JA signaling pathways (Zhang et al., 2014). In addition, SINAR is mediated by two nitrate transporters, NRT1.5 and NRT1.8, which are responsible for exporting nitrate from pericycle cells into the xylem and for retrieving nitrate from xylem sap back into root cells, respectively (Li et al., 2010; Chen et al., 2012). The expression of *NRT1.5* is repressed under Cd/Na stress or MeJA treatment, and this repression is alleviated by disruption of *EIN2 or COI1*. Conversely, *NRT1.8* expression is induced under Cd/Na stress or MeJA treatment, but induction is diminished in *erf59erf104* (*Ethylene Response Factors*) double mutants (Zhang et al., 2014). These findings indicate that ET and JA signaling modulate SINAR by downregulating *NRT1.5* and upregulating *NRT1.8.* Similar to soil stress such as Cd and Na, UV-B radiation can also induce ET production in many plants (Wang et al., 2022b). It was found that UV-B-induced nitrate reallocation from hypocotyls to roots was impaired in ET signaling mutants for *EIN2* and *EIN3* (Wang et al., 2022b). Further studies indicate that ET activates the expression of genes within the ERFs-NRT1.8 signaling module, promoting NRT1.8-mediated nitrate unloading from hypocotyl to roots (Wang et al., 2022b).

## Calcium

**Figure 2:**
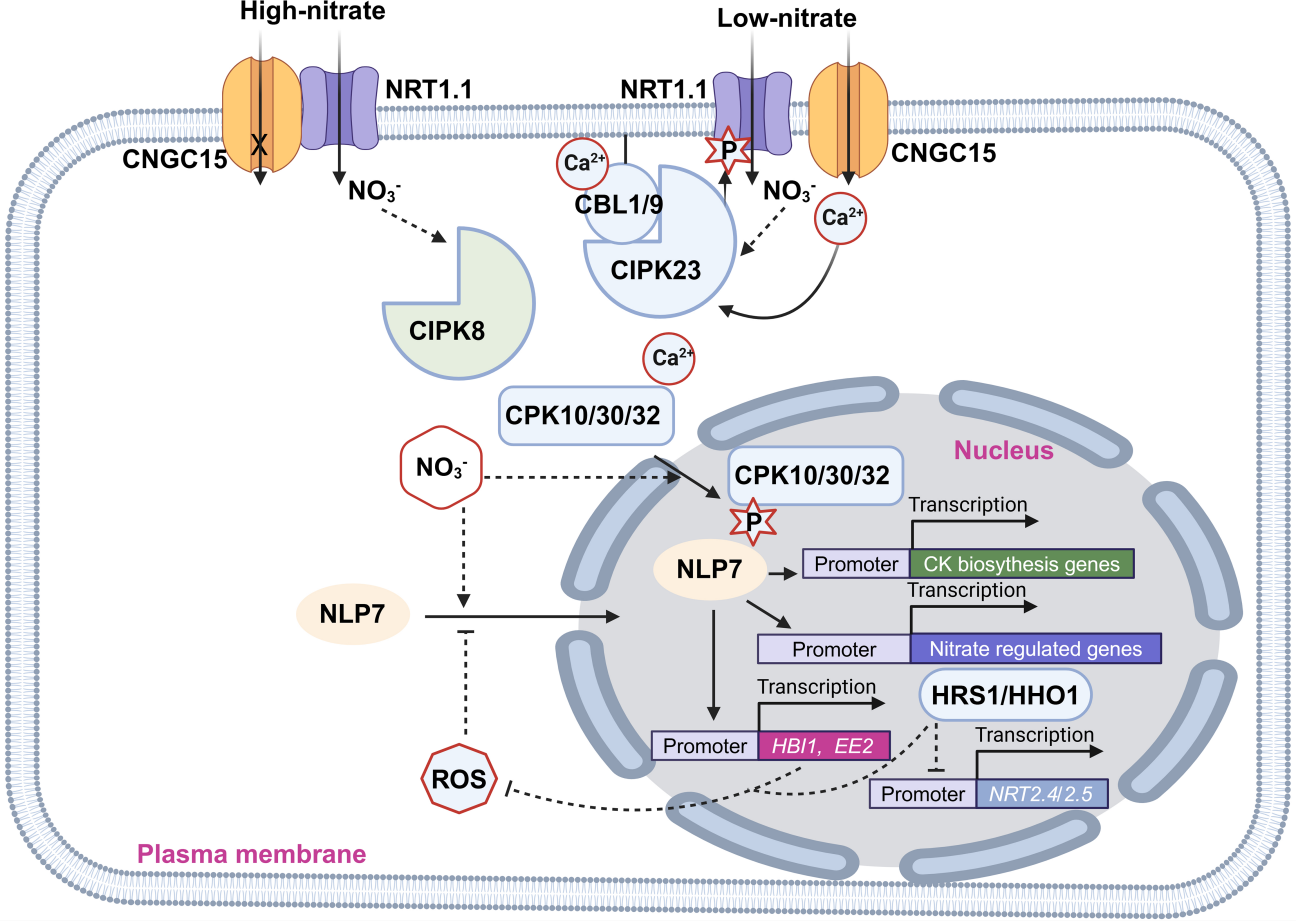
Illustration of the crosstalk between nitrate signaling and calcium as well as ROS signaling. Pointed and blunt arrowheads indicate activating and inhibiting interactions, respectively. The cross mark indicates inhibition of the channel activity of CNGC15.

Nitrate triggers Ca^2+^ fluxes in plant cells, where the resulting calcium signals transduce nitrate cues to downstream effectors, such as protein kinases and transcription factors (TFs), to regulate gene expression and adjust metabolism (illustrated in **Figure 2**) (Riveras et al., 2015). In nitrate-treated roots, cytosolic Ca^2+^ levels [Ca^2+^]_cyt_ are lower in the *nrt1.1*-AQ line (a cross of *nrt1.1* mutant and 35S::aequorin transgenic line) compared to the WT-AQ line, demonstrating that nitrate-triggered increases in [Ca^2+^]_cyt_ depend on *NRT1.1* (Riveras et al., 2015).

CNGC (Cyclic Nucleotide-gated Channel) is a class of nonselective cationic channels with permeability to monovalent and divalent cations including Ca^2+^ (Gao et al., 2016; Zhang et al., 2017). CNGC15 was reported to form a complex with NRT1.1 to control Ca^2+^ influx in a nitrate-dependent manner (Wang et al., 2021). The Ca^2+^ channel activity of CNGC15 is inhibited when it forms a heterocomplex with NRT1.1. However, upon nitrate addition, this interaction of CNGC15-NRT1.1 is weakened and the Ca^2+^ channel activity of CNGC15 is restored (Wang et al., 2021).

CBLs (Calcineurin B-like proteins) are Ca^2+^-binding proteins that interact with CIPKs (CBL-interacting protein kinases) to phosphorylate the downstream targets (Mao et al., 2023). CIPK8 and CIPK23 are inducible by nitrate. However, their induction is significantly reduced in the *nrt1.1* mutant, indicating their function within the NRT1.1-dependent signaling pathway (Ho et al., 2009; Hu et al., 2009). Notably, CIPK23 not only acts downstream in NRT1.1 signaling but also regulates NRT1.1 directly by phosphorylating it at the T101 residue, controlling both its transport and signaling functions (Ho et al., 2009). The CBL1/9-CIPK23 complex regulates the nitrate uptake activity of NRT1.1 by phosphorylating T101 residue. When T101 is phosphorylated, NRT1.1 functions as a high-affinity nitrate transporter, whereas in its dephosphorylated state, it operates as a low-affinity nitrate transporter (Liu and Tsay, 2003). In contrast, CIPK8, while less well characterized, stimulates primary nitrate responses in the low-affinity range. This role is evident from defects in *cipk8* null mutants during the low-affinity phase of nitrate-induced expression of *NRT2.1* and *NIA* genes but not in the high-affinity phase (Hu et al., 2009).

CPKs (Ca^2+^-dependent protein kinases) are Ser/Thr kinases and contain a Ca^2+^ binding domain, working for both sensing Ca^2+^ and phosphorylating targets (Schulz et al., 2013). A nitrate-sensitized genomic screen identified CPKs (Ca^2+^-sensor protein kinases) as key regulators of primary nitrate responses. CPK10, CPK30, and CPK32 phosphorylate the Ser-205 residue in the transcription factor NLP7 (NIN-like Protein 7), a master regulator of nitrate metabolism (Liu et al., 2017). NLP7 orchestrates the expression of 91 genes involved in nitrate uptake and assimilation, including *NIA1*, *NIA2*, *NRT2.1*, and *NRT2.2* (Castaings et al., 2009; Konishi and Yanagisawa, 2013; Marchive et al., 2013). Therefore, the *nlp7* mutants exhibit longer primary roots and more lateral roots, phenotypic traits characteristic of N-starved plants (Castaings et al., 2009). Nitrate induces both Ca^2+^ accumulation and CPK translocation in the nucleus, promoting the localization and phosphorylation of NLP7 in the nucleus (Liu et al., 2017). Besides, nitrate-driven activation of NLP7 fine-tunes the biosynthesis of CKs in the roots and their translocation to the shoots, where they enhance the expression of CRFs (Cytokinin Response Factors). CRFs, in turn, promote the flow of auxin by directly regulating the transcription of PIN auxin transporters, promoting the development of shoot organs (Abualia et al., 2022).

## ROS

Nitrate signaling can influence ROS production, which in turn modulates downstream processes like root nitrate uptake (illustrated in **Figure 2**) (Shin and Schachtman, 2004; Shin et al., 2005). The growth-related transcription factor *HBI1* (*Homolog of Brassinosteroid Enhanced Expression 1 Interacting with IBH1*) increases the expression levels of antioxidant genes, thereby reducing ROS accumulation in plants (Chu et al., 2021). HBI1 and its homolog *BEE2* are rapidly induced by nitrate, though this induction is dramatically reduced in *nlp7* mutants. Further analysis revealed that NLP7 directly binds to the promoters of HBI1 and BEE2, and nitrate treatment induces NLP7’s localization to the nucleus (Chu et al., 2021). These findings indicate that nitrate reduces ROS accumulation by promoting NLP7 nuclear localization, which in turn induces *HBI* expression, boosting antioxidant gene expression. Additionally, H_2_O_2_ inhibits the nitrate-promoted nuclear localization of NLP7, a process impaired in *hbi* mutants (Chu et al., 2021). Thus, these results suggest that nitrate treatment lowers H_2_O_2_ levels, while H_2_O_2_ inhibits nitrate signaling, creating a feedback loop that regulates plant growth and development (Chu et al., 2021). Furthermore, HRS1 (Hypersensitivity to Low Pi-elicited Primary Root Shortening) and its homolog HHOs are transcription factors that directly bind the promoters of two high-affinity nitrate transporters *NRT2.4* and *NRT2.5* and repress their expression (Safi et al., 2021). Besides, HHO genetic manipulations (*HRS1* overexpression and *HHO1* knockout) impair ROS accumulation in plants at early N depletion, indicating their key roles in the control of ROS accumulation in response to N starvation (Safi et al., 2021). These findings suggest that HRS1 and HHOs are also key regulators in the crosstalk between nitrate signaling and ROS signaling.

## Peptide

**Figure 3:**
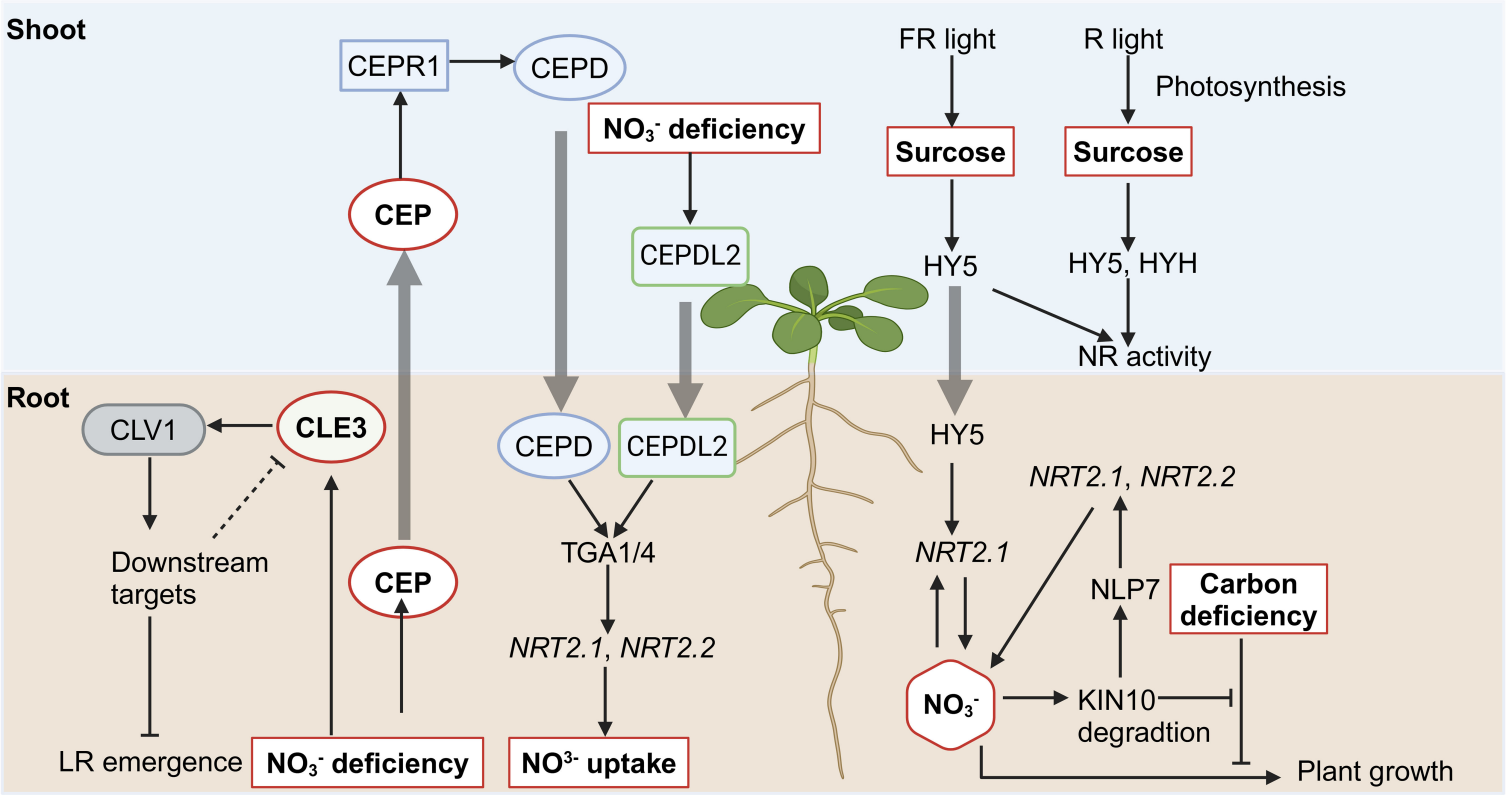
Illustration of the crosstalk between nitrate signaling and peptide as well as sucrose signaling. Pointed and blunt arrowheads indicate activating and inhibiting interactions, respectively. The thick arrow indicates the shoo-to-root or root-to-shoot translocation.

CEPs (C-terminally Encoded Peptides) are encoded in the stele of LR and are loaded into the xylem vessels to travel to shoots, where they regulate systemic nitrate acquisition responses as a ‘hunger signal’ (Tabata et al., 2014). CEPs contain 15 family members in *Arabidopsis* (Roberts et al., 2013), seven of which are rapidly and highly upregulated in root directly under low nitrate conditions (Ohkubo et al., 2017). Upon translocation, CEP peptides are perceived by the shoot-localized leucine-rich repeat receptor kinase CEPR1 (CEP Receptor 1), which is expressed in the vascular tissues of leaves (Tabata et al., 2014). The CEP-CEPR1 module upregulates the expression of nitrate transporters such as *NRT1.1*, *NRT2.1*, and *NAR2.1* (*NRT3.1*), facilitating nitrate uptake (illustrated in **Figure 3**) (Tabata et al., 2014). Since root-derived CEP peptides are recognized by CEPR1 in leaf vascular tissue and subsequently regulate the expression of nitrate transporter in roots, the systemic N-demand signaling likely involves a descending shoot-to-root signal activated downstream of CEPR1. This hypothesis led to the identification of two shoot-to-root mobile peptides, CEPD1 and CEPD2 (CEP Downstream 1 and 2), which mediate the descending signal upon the perception of root-derived CEP (Ohkubo et al., 2017). CEPDL2 (CEPD-like 2) contributes to nitrate acquisition cooperatively with CEPD1 and CEPD2, while CEPDL2 is upregulated in the leaf vasculature by the shoot nitrogen deficiency (Ota et al., 2020). In roots, CEPD1/2/CEPDL2 interact with TGA1/4 (TGACG-binding), the TFs playing a global role in root nitrate signaling. TGA1/TGA4 regulate *NRT2.1* and *NRT2.2* expression by binding to their promoters (Alvarez et al., 2014). The *tga1/4* mutants maintain basal nitrate uptake but exhibit impaired nitrate acquisition in response to shoot nitrogen demand (Kobayashi et al., 2024).

CLEs (Clavata3/Embryo Surrounding Region) peptides control meristem functions in plants (Araya et al., 2014b). CLE1, -3, -4, and -7 have been found to interact with the receptor kinase CLV1 (Clavata1), repressing LR emergence and growth under nitrogen deprivation (Araya et al., 2014a). Since *CLE* mRNAs are expressed in the pericycle while *CLV1* is expressed in phloem companion cells, therefore CLE peptides are also proposed to serve as cell–cell mobile signals, integrating N signals into root responses (Araya et al., 2014a, Araya et al., 2014b).

## Sucrose

Nitrate and sugar signaling pathways are tightly interconnected, ensuring that nitrate uptake and assimilation align with the plant’s energy status to optimize growth under varying environmental conditions (illustrated in **Figure 3**) (Fichtner et al., 2021). Sucrose availability influences nitrate-responsive gene expression and vice versa. *NRT2.1* is regulated both transcriptionally and post-transcriptionally by C- and N-derived metabolites (Lejay et al., 1999; Girin et al., 2007; Jong et al., 2014), positioning it as a key integrator in the crosstalk between nitrate and sugar pathways (Fichtner et al., 2021). Its expression is repressed in darkness but is relieved in the presence of sucrose (Lejay et al., 1999). Additionally, intermediates of the oxidative pentose phosphate pathway, such as pyruvate and shikimate, have been shown to induce the expression of *NRT2.1* (Jong et al., 2014). KIN10 (SnRK1 Catalytic Subunit Alpha Kinase 10) is identified as another key integrator in the coordination of carbon and nitrogen metabolism (Wang et al., 2022a). It was found that carbon deficiency represses the plant growth induced by nitrate, however, this inhibitory effect is relieved in the mutants of *KIN10*. Further study showed that nitrate promotes KIN10 degradation, thereby relieving the inhibitory effects of carbon deficiency on nitrate-mediated plant growth. Besides, KIN10 also phosphorylates NLP7 and promotes its cytoplasmic localization and degradation, regulating the nitrate signaling pathways. Thus, ensuring optimal nitrate signaling and the coordination of carbon and nitrogen metabolism in plants (Wang et al., 2022a).

HY5 (Long Hypocotyl 5), a core bZIP TF primarily expressed in shoots, has also been shown to move from shoots to roots upon light exposure (Chen et al., 2016). HY5 and its close homolog HYH regulate light- and sucrose-dependent nitrate reductase (NR) activity (Jonassen et al., 2008). HY5 is required for NR activation by far-red (FR) and red (R) light, as FR and R light fail to induce high NR activity in the *hy5* mutant compared to WT. In contrast, while FR light induces NR activity in the *hyh* mutant, R light does not, indicating that HYH is specifically necessary for R-induced NR activity. The *hy5hyh* double mutant exhibits reduced white light-induced NR activity compared to the single mutants, suggesting a partially redundant role for HY5 and HYH. Further insights into the role of HY5 come from studies on shoot-to-root signaling. HY5 binds directly to the promoter of *NRT2.1*, as shown by ChIP assays, and shoot illumination-induced upregulation of root *NRT2.1* is largely abolished in the *hy5* mutant (Chen et al., 2016). This HY5-mediated enhancement of root *NRT2.1* promotes nitrate uptake and depends on shoot carbon photo-assimilate (sucrose) levels. These findings underscore the role of HY5 as a systemic regulator of nitrate uptake and the carbon/nitrogen balance in response to ambient light and sucrose signals (Chen et al., 2016).

## Insights for improving NUE from nitrate signaling crosstalk

The crosstalk between nitrate signaling and other molecular pathways is crucial for optimizing plant growth, development, and stress responses. The interactions between nitrate, phytohormones, ROS, calcium, peptide, and sucrose help plants integrate environmental cues and internal nutrient status to adjust their physiological processes. Understanding these interactions provides strategies for enhancing crop NUE.

### Genome editing of phytohormone signaling for remodeling RSA

Plants exhibit diverse adaptive responses to fluctuating nitrogen availability, including optimizing their RSA under nitrogen-limited conditions to enhance nitrogen acquisition from the soil. Given the intricate crosstalk between phytohormone and nitrate signaling in RSA remodeling, genes within phytohormone signaling pathways serve as promising targets for enhancing the NUE of crops.

For instance, the QTL *DRO1* (*Deeper Rooting 1*) in *Oryza sativa*, negatively regulated by auxin, promotes deeper rooting, enhancing nitrogen uptake and cytokinin fluxes during later growth stages. The near-isogenic line bearing the allele of *DRO1* exhibited more roots within the lower soil layer of the paddy field and approximately 10% higher grain yield (Arai-Sanoh et al., 2014). *OsPIN2* plays a critical role in mediating root gravitropic responses and ensuring normal root growth angles in rice (Wang et al., 2018a). The *Os*-*pin2* mutant exhibited a large root growth angle and reduced sensitivity to gravity, leading to a shallower root system. This shallow root architecture likely enhances topsoil foraging, facilitating nutrient acquisition from the upper soil layers while also helping to avoid hypoxic conditions, ultimately promoting rice growth (Wang et al., 2018a). Therefore, *PIN2* also represents a promising genetic target for regulating NUE. In Triticum aestivum, overexpression of *TaTAR2.1-3A*, *TaTAR2.1-3B*, and *TaTAR2.1-3D*, orthologs of *AtTAR2*, enhances LR branching, as well as plant height, spike number, grain yield, and nitrogen accumulation in the aboveground parts under varying nitrogen supply levels (Shao et al., 2017).

Additionally, as previously mentioned, BSK3 is the key kinase in the BR signaling pathway that regulates root elongation. The allelic variation from Leu^319^ to Pro^319^ results enhances BR sensitivity, leading to increased root elongation, making *BSK3* a promising target for improving root growth under nitrogen-limited conditions (Jia et al., 2019). Exploiting natural allelic variants of *BSK3* or generating new variants through precise genome editing could contribute to the development of longer root systems, thereby enhancing nitrate uptake under low nitrogen conditions. These studies illustrate how modulating phytohormone signaling can affect RSA to improve NUE, offering valuable insights into the phytohormone-mediated RSA-NUE relationship in crops.

### Application of synthetic chemicals for remodeling RSA

The application of synthetic chemicals for remodeling RSA involves the use of chemical agents to modify root overall root system development, enhancing the NUE of plants. For instance, foliar application of NAA increased the endogenous IAA content, enhanced the activity of enzymes related to nitrogen assimilation, and boosted the rate of photosynthesis. In addition, NAA promoted root activity, regulated root morphology and structure, and facilitated further nitrogen uptake and plant growth (Jiang et al., 2024). In addition, treatment with ethephon (a direct source of ET) and ACC (an ET precursor) promotes the initiation of LR and enhances nitrogen absorption (Liu et al., 2010). The application of GA3 stimulates cell division and elongation in the root meristem, resulting in longer PR and LR, thereby increasing the nitrogen uptake of maize (Ullah et al., 2022).

Hormones also intersect with CEP receptor signaling to regulate RSA in plants (Chapman et al., 2020). CEP peptides act not only as “N-hunger signals” that are perceived by receptors in the shoot, triggering further signaling that induces the expression of nitrate transporters in the roots, but also interact with hormone signaling, particularly cytokinin, to promote shallower LR growth (Chapman et al., 2024). Furthermore, CEPs have been shown to repress auxin biosynthesis and alter auxin transport in the roots of *Medicago* and *Arabidopsis*, affecting gravitropic responses (Chapman et al., 2020). Based on these findings, there have been efforts to exploit synthetic CEPs to enhance nitrate uptake. One study reported that applying synthetic CEP peptides significantly boosted nitrate uptake in both *Medicago* and *Arabidopsis* by 70–140% under low nitrate conditions, by modulating the transcription of nitrate transporter genes (Roy et al., 2021).

### Carbon–nitrogen metabolism regulation

The crosstalk between sugar and nitrate signaling highlights the close interconnection between carbon and nitrogen metabolism (Aluko et al., 2023). Therefore, genes involved in carbon metabolism also represent promising targets for enhancing NUE. A moderate nitrogen supply can reduce the expression level of *OsSTP28* (*Sugar Transporter Protein 28*), a sugar transporter that negatively regulates nitrogen response during rice tillering, thereby increasing the glucose concentration in the apoplast of the stem base (Zhang et al., 2024). This process inhibits the activity of the transcription factor *OSH15* (*Oryza Sativa Homeobox 15*) by altering the methylation status on histone H3K27 (Histone H3 Lysine 27) and activates the GA degradation pathway, primarily driven by the gibberellin oxidase GA2oxs, which ultimately leads to increased tiller number and higher yield. In addition, the study uncovered a more effective allelic variant of *OsSTP28*, which enhances modern cultivated rice’s response to nitrogen more efficiently, promoting tillering and ultimately improving yield (Zhang et al., 2024). This provides valuable genetic resources for improving both high yield and NUE in rice.

Recently, two novel carbon-nitrogen metabolism-coupled photorespiratory bypasses were synthesized in rice, which were successfully assembled and expressed efficiently in chloroplasts without releasing carbon dioxide (Chen et al., 2025). The introduction of these bypasses could convert glycolate metabolism in the chloroplasts into glyoxylate, thereby promoting the synthesis of amino acids, energy, and carbohydrates. This, in turn, significantly enhanced both photosynthetic efficiency and NUE in rice. Field trials demonstrated that rice with the carbon-nitrogen metabolism-coupled photorespiratory bypass exhibited a 19.0% yield increase under normal growth conditions compared to the wild-type control, and up to a 44.1% increase under low nitrogen conditions (Chen et al., 2025). This research not only uncovers a new mechanism for regulating carbon-nitrogen metabolism through photorespiratory bypasses but also provides a scientific basis for developing high-yield, fertilizer-efficient, and stress-tolerant rice varieties.

### SINAR engineering to improve NUE under stress

As previously described, ET/JA and NRT1.5/1.8-involved SINAR promote the stress tolerance of plants under cadmium and salt (Zhang et al., 2018). However, reduced nitrate root-to-shoot translocation means low NUE and inhibited plant development, resulting in the trade-off between plant growth and stress tolerance. Recently, with the development of gene-editing techniques and a more comprehensive understanding of SINAR, genetic engineering of SINAR for the most appropriate shoot/root nitrate ratio is promising to improve the NUE of plants in adverse environments.

Specifically, genes involved in root-to-shoot transport can be targeted through genetic engineering, such as *NRT1.5*, which exports nitrate from pericycle cells into the xylem (Lin et al., 2008), and *NRT1.8*, which retrieves nitrate from xylem sap back into root cells (Li et al., 2010). Under salt and Cd^2+^ treatment, the expression of *NRT1.8* for xylem nitrate retrieval is upregulated, while the expression of *NRT1.5* for xylem loading is downregulated, resulting in increased SINAR to roots in response to these stressors (Zhang et al., 2014). Besides, *NPF2.3*, co-expressed in pericycle cells with *NRT1.5*, mediates xylem nitrate loading, particularly under salt stress when *NRT1.5* is downregulated (Taochy et al., 2015). Thus, the gene expression alteration or key amino acid substitution of these nitrate transporters could be precisely edited to appropriately regulate the nitrate root-to-shoot ratio. Additionally, considering NRT1.5/1.8-involved SINAR is under the regulation of ET/JA phytohormones, involved genes in the ET/JA signaling module could also be considered to engineer SINAR (Zhang et al., 2017).

Within plant cells, excessive nitrate can be stored in the vacuole, enabling plants to regulate nitrogen levels, avoid toxicity, and maintain a nitrate reservoir for future needs. However, some nitrate-inefficient genotypes exhibit reduced efficiency in remobilizing nitrate into the cytoplasm, leading to excessive nitrate accumulation in the vacuole. This phenomenon is considered “luxury consumption” under conditions of abundant nitrate supply in modern agricultural systems. Studies have shown that reduced root VSC (Vacuolar Sequestration Capacity) of nitrate in the high-NUE genotype enhances nitrate transport to shoots compared to the low-NUE genotype, thereby promoting NUE in *Brassica napus* (Han et al., 2016). This effect is likely mediated by the upregulation of *BnNRT1.5*, downregulation of *BnNRT1.8*, and inhibition of tonoplast proton-pumps activities (Han et al., 2016). This presents an additional strategy to improve NUE through SINAR editing. Furthermore, the CLCa (Chloride Channel a) is the main 2NO_3_^−^/1H^+^ exchanger responsible for vacuolar nitrate accumulation (De Angeli et al., 2006). A mutation in a glutamate residue at position 203 (CLCa_E203A_) converts the 2NO_3_^−^/1H^+^ exchanger into a NO_3_^−^ channel (Hodin et al., 2023). When introduced into a *clca* knockout mutant, this mutation resulted in impaired nitrate accumulation and enhanced NUE compared to both the wild-type and *clca* mutant (Hodin et al., 2023). Therefore, the root-specific expression of CLCa_E203A_ could also enhance NUE without the growth disruptions caused by *CLCa* expression in shoots.

## Concluding remark

The molecular mechanisms governing nitrate nutrition in plants constitute a highly intricate network involving specialized nitrate transporters and signaling molecules. These components work in concert to ensure the efficient uptake, systemic distribution, and metabolic assimilation of nitrate, enabling plants to adapt dynamically to fluctuations in environmental nitrogen levels. This regulatory network not only detects nitrate availability but also coordinates a variety of physiological and developmental processes, such as root architecture remodeling, shoot growth regulation, and metabolic adjustments, all aimed at optimizing nitrogen acquisition and utilization. In this review, we provide a comprehensive analysis of the crosstalk between nitrate and signaling molecules, exploring their direct and indirect effects on NUE and related processes in the model plant *Arabidopsis.* Establishing a strong link between these signaling molecules and the enhancement of crop NUE is essential for advancing research aimed at improving both NUE and crop yield. Recent breakthroughs in genome editing technologies, such as CRISPR/Cas, offer precise tools for modifying genes involved in nitrate signaling. We anticipate that further discoveries of nitrogen-efficient genes, influenced by the intricate interactions between nitrate and signaling molecules, will pave the way for developing crop varieties with improved NUE.
